# Systemic AAV-h*GCDH* Gene Therapy Alleviates Glutaric Acid Accumulation and Attenuates Chronic Brain Vacuolation in a Novel Mouse Model of Glutaric Aciduria Type I

**DOI:** 10.3390/ijms27125569

**Published:** 2026-06-20

**Authors:** Su Jin Kim, Yu Hwa Nam, Eun Young Joo, Jisun Park, Saeyoung Park, Sung-Chul Jung, Dong-Kyu Jin

**Affiliations:** 1 Department of Pediatrics, Inha University Hospital, Inha University College of Medicine, Incheon 22332, Republic of Korea; kimsjped@inha.ac.kr (S.J.K.); freegana@inha.ac.kr (E.Y.J.); pedendo@inha.ac.kr (J.P.); 2 Department of Biochemistry, College of Medicine, Ewha Womans University, Seoul 07804, Republic of Korea; queennnam@naver.com (Y.H.N.); saeyoung@ewha.ac.kr (S.P.); 3 School of Medicine, Sungkyunkwan University, Suwon 16419, Republic of Korea

**Keywords:** glutaric aciduria type 1, glutaryl-CoA dehydrogenase, adeno-associated virus, gene therapy, neurometabolic disease, CRISPR/Cas9 mouse model

## Abstract

Glutaric aciduria type 1 (GA1) is a rare neurometabolic disorder caused by glutaryl-CoA dehydrogenase (GCDH) deficiency, leading to the accumulation of neurotoxic metabolites that can cause both acute encephalopathic crises and progressive, insidious brain injury. Current management primarily relies on a protein-restricted diet, which remains therapeutically insufficient and burdensome for patients, highlighting the need for disease-modifying therapies. In this study, we established a novel GA1 mouse model using CRISPR/Cas9 technology and evaluated the preclinical efficacy of systemic recombinant adeno-associated virus (rAAV)-mediated gene therapy. Under standard dietary conditions without high-lysine challenge, our GA1 model exhibited sustained cerebral and hepatic glutaric acid (GA) accumulation and distinct chronic vacuolation in the hippocampus and cerebellum, mirroring the insidious-onset GA1 phenotype. Five-week-old mice received a single intravenous injection of rAAV-h*GCDH* using either rAAV2/8 or rAAV2/9 serotypes. Systemic rAAV-mediated gene therapy significantly reduced GA accumulation and attenuated chronic neuropathological changes in this GA1 mouse model for both serotypes. Our findings support the hypothesis that peripheral metabolic correction may play an important role in preventing the chronic neuropathological changes associated with *GCDH* deficiency. However, further investigation using tissue-specific expression systems is required to definitively delineate the relative contributions of hepatic versus central *GCDH* restoration to the observed neuroprotection.

## 1. Introduction

Glutaric aciduria type 1 (GA1) is a rare neurometabolic disorder caused by mutations in the *GCDH* gene [1,2], which encodes glutaryl-CoA dehydrogenase (*GCDH*), a key enzyme in the catabolic pathways of L-lysine, L-hydroxylysine, and L-tryptophan. *GCDH* deficiency leads to the systemic accumulation of neurotoxic metabolites—particularly glutaric acid (GA), 3-hydroxyglutaric acid (3-OH-GA), and glutarylcarnitine (C5DC)—in body tissues, especially the brain. These metabolites induce striatal damage through excitotoxic mechanisms, classically manifesting as acute encephalopathic crises triggered by catabolic stress [3]. However, insidious progressive neurological symptoms such as dystonia and long-term striatal injury are increasingly recognized in patients due to chronic exposure to these neurotoxins, even in the absence of acute metabolic crises [3,4]. Although the implementation of newborn screening (NBS) and early dietary management has improved clinical outcomes [5,6], these conservative treatments—such as a lysine-restricted diet and L-carnitine supplementation—are preventative rather than curative. These therapies demand lifelong strict adherence, severely impacting the quality of life for patients and families [7,8]. Therefore, there is an unmet need for disease-modifying treatments that correct the underlying enzyme defect within a precision medicine framework [9].

Adeno-associated virus (AAV)-mediated gene replacement therapy has emerged as a promising curative strategy for monogenic metabolic disorders such as GA1. Previous preclinical studies have shown that recombinant AAV-mediated human *GCDH* cDNA (rAAV-h*GCDH*) delivery can restore enzyme activity and prevent neurodegeneration in *Gcdh*-deficient mice [10,11]. However, selecting the optimal capsid remains a critical challenge to effectively address both the systemic metabolic defects and neurological manifestations [12]. In this study, we selected rAAV2/8 and rAAV2/9 as comparative delivery vectors due to their distinct and well-characterized tissue tropisms. AAV8 exhibits strong hepatotropic transduction and has been widely utilized in liver-directed gene therapy [13], whereas AAV9 possesses the unique capability to penetrate the blood–brain barrier (BBB) and achieve widespread central nervous system (CNS) transduction following systemic administration [14]. Given that GA1 is a systemic organic aciduria with devastating neurological consequences, comparing a primarily hepatotropic vector (AAV8) with a highly neurotropic, BBB-permeable vector (AAV9) provides a comprehensive cross-platform evaluation.

The *Gcdh*-deficient mouse described by Koeller et al. recapitulates key disease features, including characteristic biochemical abnormalities, striatal degeneration, and motor deficits [15]. However, many subsequent therapeutic studies have used a high-lysine diet (HLD) challenge to induce acute metabolic decompensation and exacerbate the phenotype for validation [10,11,16]. Unlike those studies, our study focuses on chronic neuropathology under physiological dietary conditions.

In this study, we compared systemic rAAV2/8- and rAAV2/9-mediated *GCDH* gene delivery in a newly generated GA1 mouse model using CRISPR/Cas9 technology to evaluate their relative therapeutic efficacy and to explore the contribution of peripheral metabolic correction versus CNS transduction to disease modification.

## 2. Results

### 2.1. Establishment and Characterization of the GA1 Mouse Model

To confirm the successful generation of the GA1 mouse model, we conducted polymerase chain reaction (PCR) analysis on offspring from the CRISPR/Cas9-edited founder line #4. Compared with wild-type (WT) littermates, this analysis consistently demonstrated the expected gene truncation in the target region, verifying the establishment of a stable *GCDH* null genotype.

**Figure 1:**
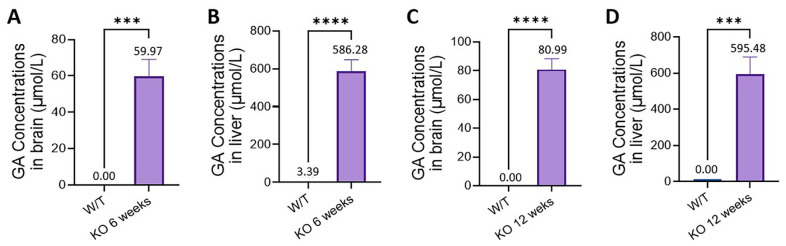
Mean GA concentrations in brain (**A**) and liver (**B**) tissues at 6 weeks of age and, in brain (**C**) and liver (**D**) tissues at 12 weeks of age. The statistical analyses were performed using Student’s *t*-test (*** *p* < 0.001, and **** *p* < 0.0001).

To characterize the GA1 mouse model, we first assessed the clinical phenotype of GA1 mice maintained on a standard diet. Consistent with reports on other *Gcdh*-deficient mice [15], our GA1 mice exhibited no overt neurological symptoms—such as seizures, paralysis, or significant motor deficits—and showed no reduced survival or differences in body weight gain compared to WT controls up to 12 weeks of age. Despite the absence of a severe clinical phenotype, the biochemical analysis revealed a profound metabolic defect characteristic of human GA1. Using our validated liquid chromatography-tandem mass spectrometry (LC-MS/MS) method, we quantified the GA levels across various biological samples; GA1 mice displayed markedly elevated GA concentrations in liver and brain tissues compared to WT mice, which had negligible levels (Figure 1). This biochemical accumulation was evident as early as 6 weeks of age and persisted through 12 weeks. These results confirm that our CRISPR/Cas9-generated GA1 mice model faithfully recapitulates the biochemical hallmark of human GA1, providing a robust platform for evaluating gene therapy efficacy via metabolite reduction.

### 2.2. Production and In Vitro Validation of rAAV-hGCDH Vectors

**Figure 2:**
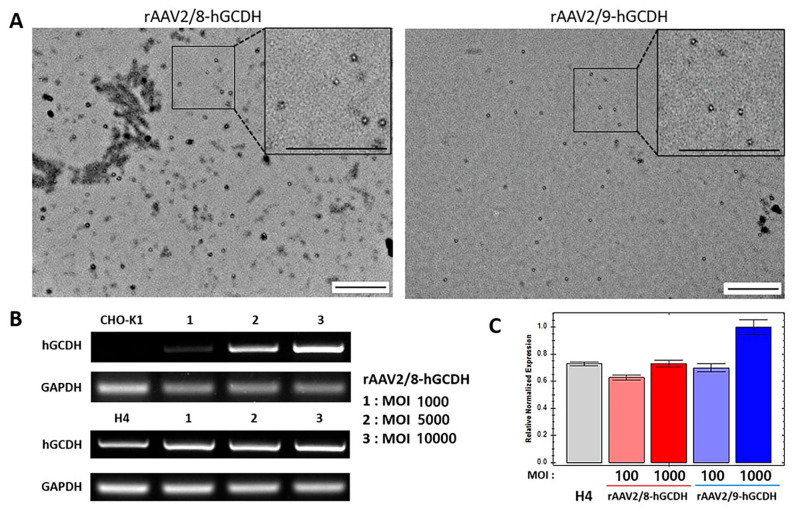
rAAV-h*GCDH* production. (**A**) Observation of produced rAAV-h*GCDH* via transmission electron microscope. Representative and enlarged images observed for each experimental group (rAAV2/8-h*GCDH* and rAAV2/9-h*GCDH*) presented. Scale bars indicate 1 μm. CHO-K1 and H4 cells were infected with rAAV-h*GCDH* at various MOIs (multiplicities of infection), and h*GCDH* expression was confirmed using RT-PCR (**B**) and qPCR (**C**).

Prior to in vivo studies, we confirmed the quality and potency of the viral vectors. Transmission electron microscopy (TEM) revealed that both rAAV2/8-h*GCDH* and rAAV2/9-h*GCDH* particles exhibited typical icosahedral morphology and high purity (Figure 2A). To evaluate the transgene expression, we transduced CHO-K1 and H4 cells with the vectors at various multiplicities of infection (MOIs). Reverse Transcription Polymerase Chain Reaction (RT-PCR) and Real-time Quantitative PCR (qPCR) analyses showed a dose-dependent increase in human *GCDH* mRNA levels in both cell lines, demonstrating that the vectors effectively transduce cells and drive therapeutic gene expression in vitro (Figure 2B,C).

### 2.3. Biodistribution and Transgene Expression In Vivo

**Figure 3:**
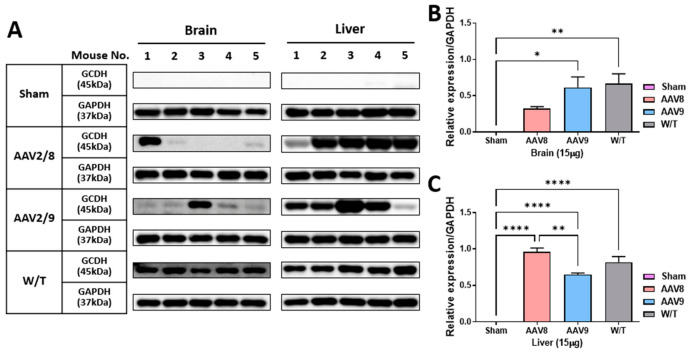
The h*GCDH* protein expression confirmed after rAAV-h*GCDH*s injection. (**A**) Expression of h*GCDH* in brain and liver tissues of mice at 12 weeks according to Western blot analysis. The expression in brain (**B**) and liver (**C**) was quantified using Image J software (Version 1.49), and its level was normalized to that of GAPDH. One-way ANOVA and Tukey’s post hoc test were performed for comparison between groups. The data are presented as the mean ± SEM (* *p* < 0.05; ** *p* < 0.01; **** *p* < 0.0001).

To confirm the successful delivery and expression of the therapeutic transgene, we performed qPCR and Western blot analyses on tissues harvested at 12 weeks post-injection. qPCR analysis of genomic DNA revealed substantial copies of the human *GCDH* gene in the livers of mice treated with rAAV2/8-h*GCDH* and rAAV2/9-h*GCDH*. Viral genome copies were also detected in the brain, indicating that systemic administration enabled viral distribution to the CNS. While the endogenous *GCDH* protein was completely absent in tissues from phosphate-buffered saline (PBS)-treated GA1 mice, both AAV vectors successfully restored *GCDH* protein expression. Notably, the hepatic *GCDH* levels in treated mice were comparable to or exceeded those in WT mice (Figure 3).

### 2.4. Therapeutic Efficacy of Systemic rAAV-hGCDH Administration

#### 2.4.1. Improvement in Metabolite Accumulation

**Figure 4:**
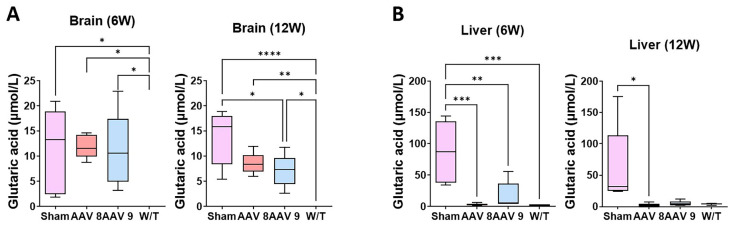
Measurement of the GA concentration in the brain (**A**) and liver (**B**) at 6 and 12 weeks after rAAV-h*GCDH* injection. One-way ANOVA and Tukey’s post hoc test were performed for comparison between groups. The data are presented as the mean ± SEM (* *p* < 0.05; ** *p* < 0.01; *** *p* < 0.001; **** *p* < 0.0001). GA, glutaric aciduria type I; AAV, adeno-associated virus; GCDH, glutaryl-CoA dehydrogenase.

To determine whether the restored *GCDH* expression achieved metabolic correction, we quantified the GA levels in brain and liver tissues. In the brain—where metabolite accumulation drives neurotoxicity—the sham group exhibited substantial GA buildup, whereas rAAV2/8-h*GCDH* and rAAV2/9-h*GCDH* treatments significantly reduced the cerebral GA levels (Figure 4A). In the liver, the primary site of *GCDH* metabolism, both serotypes normalized GA concentrations to levels statistically indistinguishable from those of WT controls, reflecting efficient transduction and enzymatic restoration (Figure 4B). The brain GA reduction highlights systemic AAV gene therapy’s potential to mitigate GA-associated neurotoxicity. Taken together, these data demonstrate the potential for the systemic administration of rAAV-h*GCDH* to alleviate core metabolic defects and attenuate the accumulation of toxic metabolites in the systemic tissue and CNS in GA mice on a standard diet. This potent metabolic improvement effect persisted during the 12-week observation period, suggesting the potential for long-term efficacy from a single systemic administration.

#### 2.4.2. Histological Protection in the Brain

To assess whether the metabolic mitigation translated into structural neuroprotection, we performed hematoxylin and eosin staining on brain sections from all groups.

**Figure 5:**
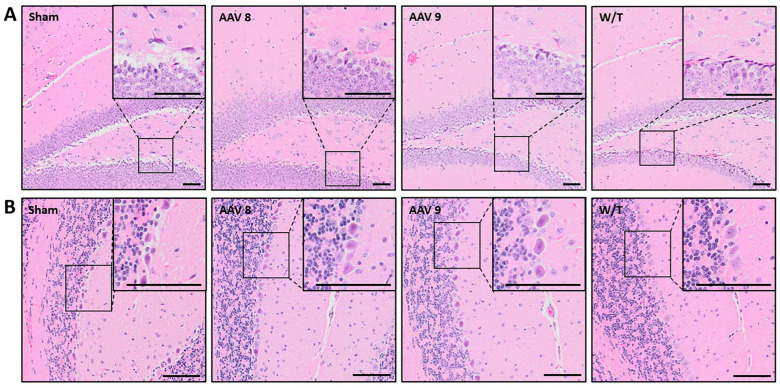
H&E staining of brain tissue at 12 weeks after rAAV-h*GCDH* injection. Representative images observed for the hippocampus (**A**) and cerebellum (**B**), as well as enlarged images in each group. Scale bars indicate 200 μm.

1. Hippocampus; In the sham-treated GA1 mice, distinct vacuolation was evident in the hippocampal region, indicating structural degeneration due to toxic metabolite accumulation. In contrast, AAV8- and AAV9-treated mice exhibited preserved tissue architecture without vacuolation, closely resembling the wild-type controls (Figure 5A).
2. Cerebellum; The sham-treated mice also showed pathological changes in the cerebellum, including irregular nuclear morphology (e.g., nuclear fusion and uneven shapes) accompanied by vacuolation. However, the AAV8- and AAV9-treated groups displayed nuclei of regular size and shape with no vacuolation, which were indistinguishable from the WT controls (Figure 5B).

These histological findings demonstrate that systemic AAV-h*GCDH* gene therapy effectively attenuates GA1-associated microscopic brain pathology.

## 3. Discussion

Although the integration of GA1 into NBS has significantly improved clinical outcomes, the disease remains a challenging neurometabolic disorder [5]. While early dietary and emergency management during acute encephalopathic crises have significantly reduced severe neurological sequelae, a substantial number of patients still develop progressive neurological impairment even in the absence of overt metabolic decompensation [17]. This clinical reality underscores the unmet need for disease-modifying interventions that can be applied during the latency phase to prevent cumulative brain injury.

In this study, we established a novel GA1 mouse model using CRISPR/Cas9 technology that effectively recapitulates the insidious-onset phenotype of human GA1. A key distinction of our model compared to the widely used Koeller et al. model is its ability to manifest chronic neuropathology under standard dietary conditions [15]. Previous studies have provided invaluable insights into preventing acute metabolic decompensation. However, most relied on HLD challenges to induce acute striatal injury and therefore primarily modeled the acute-onset phenotype [10,11,18]. In contrast, our model exhibited persistent GA accumulation and distinct vacuolation in the hippocampus and cerebellum without external dietary stress [16]. Notably, despite marked tissue GA accumulation and histopathological abnormalities, our GA1 mice did not exhibit overt motor or behavioral deficits under a standard diet (Figure S1). This observation is consistent with the clinical presentation of insidious-onset GA1 patients. Patients with this phenotype often lack a history of acute encephalopathic crises and may remain clinically asymptomatic for prolonged periods, despite the presence of progressive brain injury detected by neuroimaging [3,17]. Taken together, these findings suggest that our model may better recapitulate the chronic and progressive aspects of GA1 that are not fully captured by acute challenge models. As such, it provides a useful platform for investigating mechanisms of chronic neurotoxicity and for evaluating preventive therapeutic strategies aimed at long-term disease modification.

Previous gene therapy studies for GA1 have explored both systemic and CNS-directed AAV-mediated *GCDH* delivery strategies, demonstrating varying degrees of metabolic correction and neurological benefit [10,11,18]. Li et al. demonstrated that intracerebroventricular (ICV) delivery of AAV vectors effectively rescued striatal neurons and improved survival in *Gcdh*-deficient mice challenged with an HLD [11]. While CNS-directed approaches offer high local transduction efficiency, they are invasive and technically challenging for widespread clinical application. Currently, the focus is shifting towards systemic administration. Previously, the flux of GA and 3-OH-GA across the BBB was considered limited; intracerebral production of these metabolites might be the primary driver of neurotoxicity [19]. However, Barzi et al. challenged this view through serial liver transplantation experiments, demonstrating that toxic metabolites originating in the liver significantly contribute to striatal damage [18]. This finding provides a rationale that liver-targeted or systemic gene therapies can be a viable strategy to prevent brain injury. A recent study by Mateu-Bosch et al. demonstrated that the systemic delivery of AAV-*GCDH* significantly ameliorated the disease manifestations, improved survival, and preserved motor function in HLD-induced GA1 mice, particularly when treatment was administered after the rapid growth phase [10]. In contrast, neonatal treatment failed to sustain a long-term therapeutic benefit, because liver growth diluted the vector genomes and reduced hepatic *GCDH* expression below the therapeutic threshold. These findings underscore the importance of treatment timing for maintaining effective hepatic transgene expression. Consistent with this concept, we administered AAV vectors to 5-week-old mice, thereby avoiding the period of most rapid liver growth and minimizing early vector dilution. Under these conditions, therapeutic efficacy was maintained throughout the 12-week observation period, with a sustained reduction in GA accumulation and attenuation of chronic neuropathological changes. However, although our results suggest an improved persistence of therapeutic benefit compared with neonatal treatment paradigms, the current study was not designed to evaluate the long-term durability beyond the observation period. Therefore, extended longitudinal studies will be necessary to determine whether transgene expression and metabolic correction remain stable throughout adulthood and aging.

Recent advances in AAV capsid engineering have yielded novel CNS-targeted vectors, including retrograde AAV11 [20], locally efficient AAV13-7m8 variants [21], and BBB-penetrating AAVhu.32-PLUS [22], which demonstrate enhanced neurotropism and reduced hepatotropism. These next-generation AAV vectors may further improve the therapeutic window of gene therapy for neurometabolic disorders by enhancing CNS delivery while reducing off-target exposure and vector dose requirements. Nevertheless, AAV8 and AAV9 remain among the most extensively characterized and clinically relevant serotypes for systemic gene transfer. AAV8 has demonstrated clinical improvement and long-term expression stability by restoring factor IX levels in hemophilia B patients through robust hepatic transduction [23], while AAV9 has established itself as a leading platform for CNS-targeted gene therapy through the success of systemic gene-replacement therapy for spinal muscular atrophy [24]. In this study, AAV8 and AAV9 were selected because they provide distinct tissue tropisms relevant to the pathophysiology of GA1. Despite these distinct biodistribution profiles, both serotypes demonstrated comparable efficacy in attenuating chronic vacuolation in the hippocampus and cerebellum and reducing cerebral GA levels in our model. These results indicate that peripheral metabolic correction plays a pivotal role in neuroprotection, although the respective contributions of hepatic versus CNS-specific *GCDH* restoration require further elucidation. However, interpretation of these findings should be made with caution. Although AAV8 is predominantly hepatotropic, and AAV9 exhibits broader CNS transduction following systemic administration, viral genomes were detected in both the liver and brain after treatment with each vector. Therefore, the present study cannot definitively distinguish the relative contributions of hepatic metabolic correction and direct CNS *GCDH* restoration to the observed neuroprotective effects. Furthermore, quantitative biodistribution analyses across additional organs and cell-type-specific transduction studies were not performed. Future investigations incorporating detailed biodistribution profiling and cellular-level transduction analyses will be important to clarify the mechanisms underlying the therapeutic efficacy and to define the respective roles of peripheral and CNS correction in GA1.

Despite the promising findings, our study has several limitations. Although therapeutic efficacy was maintained throughout the 12-week follow-up period after treatment at 5 weeks of age, the long-term durability of transgene expression and metabolic correction remains unknown. This question is particularly relevant for pediatric applications of liver-directed AAV gene therapy, where continued liver growth, age-dependent changes in hepatocyte turnover, and long-term vector persistence may influence the sustained therapeutic benefit. Accordingly, future studies with substantially longer observation periods will be required to determine whether the biochemical and neuropathological improvements observed in the present study can be maintained throughout the lifespan. An additional consideration for the clinical translation of AAV-mediated gene therapy is the host immune response against viral capsids and transgene products [25]. Although no overt adverse effects were observed during the 12-week study period, comprehensive toxicological assessments were not performed in the present study. Serum liver enzyme measurements, hematological analyses, inflammatory cytokine profiling, and histopathological evaluation of non-target organs would provide important information regarding the safety profile of systemic AAV-mediated gene therapy and should be incorporated into future preclinical studies. Pre-existing or treatment-induced neutralizing antibodies against AAV capsids may reduce the transduction efficiency and present a significant barrier to vector re-administration [26]. Furthermore, high systemic AAV doses have been associated with hepatic toxicity and immune activation in both preclinical and clinical studies [27]. Future optimization of AAV-*GCDH* therapy should focus not only on improving the efficacy but also on expanding the therapeutic safety window for pediatric metabolic disease. Recent reviews of emerging gene delivery platforms emphasize that successful clinical translation requires the coordinated optimization of tissue targeting, vector dose, immunogenicity, payload design, and long-term persistence [28,29]. In this context, several strategies may be relevant for GA1, including capsid engineering to enhance liver or CNS specificity, promoter optimization to restrict *GCDH* expression to therapeutically relevant tissues, dose reduction strategies to minimize systemic exposure, and improved vector designs that reduce off-target transduction and immune activation. Although alternative non-viral delivery platforms are rapidly advancing, AAV vectors currently remain among the most clinically established systems for achieving sustained in vivo transgene expression. Therefore, future AAV-*GCDH* development should incorporate detailed biodistribution, toxicology, immunogenicity, and long-term persistence studies to define a safer and more durable therapeutic strategy.

Another limitation of this study is the exclusion of striatal histological analysis. Classically, GA1 is highly associated with striatal injury, which drives the severe dystonic-dyskinetic movement disorders seen in patients [3]. However, the preclinical literature indicates that extensive striatal necrosis in *Gcdh*-deficient mice is a feature tightly linked to acute encephalopathic crises triggered by HLD challenges [15,16]. Unlike acute metabolic crisis models that predominantly exhibit striatal degeneration, our model demonstrates chronic neuropathological alterations under standard dietary conditions, with the most prominent abnormalities observed in the hippocampus and cerebellum. Consequently, we focused our therapeutic evaluation on the hippocampus and cerebellum, further investigations under hHLD will be needed to definitively confirm whether this approach offers equal protection against acute striatal injury.

The lack of direct *GCDH* enzyme activity assays and the reliance on GA measurements as the primary indicator of metabolic correction represent additional limitations of this study. Although GA is the major metabolite accumulating as a consequence of *GCDH* deficiency, additional disease-relevant biomarkers, including 3-OH-GA, C5DC, and other carnitines, were not evaluated. Future studies incorporating broader metabolomic profiling will be important to further characterize the extent of metabolic correction achieved by AAV-mediated *GCDH* gene therapy and to better understand the relationship between biochemical rescue and neuropathological outcomes. Furthermore, while deletion-specific PCR demonstrated successful generation of the targeted *Gcdh* allele, sequencing across the deletion junction was not performed in the present study. Such sequence-level validation would further strengthen the molecular characterization of the CRISPR/Cas9-generated model and should be incorporated in future studies.

Finally, since our model displayed a mild clinical phenotype without overt motor deficits, behavioral assessments were limited. In addition, we could not perform in vivo magnetic resonance spectroscopy to monitor real-time, dynamic changes in cerebral metabolites (e.g., NAA recovery) within the same subject. Comprehensive neurocognitive testing in future research could provide deeper insights into the functional correlation of the histological neuroprotection observed in this study.

In conclusion, we established a novel CRISPR/Cas9-generated *Gcdh*-deficient mouse model that recapitulates key biochemical and neuropathological features of insidious-onset GA1 under standard dietary conditions. Systemic AAV-mediated *GCDH* gene therapy significantly reduced GA accumulation and attenuated chronic vacuolar pathology in the hippocampus and cerebellum. Although AAV8 and AAV9 possess distinct tissue tropisms, both vectors produced comparable therapeutic benefits in this model. These findings support the hypothesis that peripheral metabolic correction may contribute substantially to neuroprotection in GA1, while highlighting the need for future studies to define the relative contributions of hepatic and CNS *GCDH* restoration. Collectively, our results provide further support for the therapeutic potential of systemic AAV-mediated gene replacement as a treatment strategy for GA1.

## 4. Materials and Methods

### 4.1. Generation of a GCDH Knock-Out Mouse Using CRISPR/Cas9

A novel *Gcdh* knock-out (KO) mouse model was generated using the CRISPR/Cas9 system. The target deletion spanned the region from the intron to exon 2, including the ATG start codon of the *Gcdh* gene, thereby effectively disrupting the gene function (Figure S2). The detailed generation process, including reagent synthesis and founder generation, was performed by Macrogen Inc. (Seoul, Republic of Korea). Briefly, Cas9 mRNA and single-guide RNAs (sgRNAs) targeting the *Gcdh* gene were microinjected into zygotes according to the manufacturer’s instructions. The resulting F0 founder mice were genotyped at three weeks of age using T7 endonuclease I (T7E1) assays and PCR analysis of the target site. Genotyping confirmed mutations in all 21 founder mice. Two strains were initially established, but line #4 was selected for further breeding and experiments owing to its favorable growth characteristics and the genetic stability of its offspring (Figure S3).

### 4.2. Development and Validation of Biochemical Analysis

Quantification of GA in biological samples was performed using liquid chromatography-tandem mass spectrometry (LC-MS/MS), based on a modification of the previously described methods [30,31]. The method was validated for selectivity, linearity, accuracy, and other parameters, all of which met the acceptance criteria. Detailed validation data and chromatograms are provided in the Supplementary Materials (Figure S4).

### 4.3. AAV Vectors Production

#### 4.3.1. Construction of the pAAV-h*GCDH* Plasmid

Human *GCDH* cDNA (h*GCDH*) was synthesized from mRNA isolated from HepG2 cells and verified via Sanger sequencing. This cDNA was cloned into an AAV transfer vector featuring a cytomegalovirus enhancer/chicken β-actin promoter, woodchuck hepatitis virus post-transcriptional regulatory element, human growth hormone polyA signal, and AAV serotype 2 inverted terminal repeats. Transcriptional activity of the resulting pAAV-h*GCDH* plasmid was confirmed via RT-PCR after transfection into CHO-K1 cells (Figure S5).

#### 4.3.2. Production and Purification of Recombinant AAV Vectors

Recombinant AAV (rAAV) vectors were produced in HEK293T (ATCC, CRL-3216, USA) cells via a triple-transfection method with calcium phosphate precipitation [32,33,34]. Cells were co-transfected with the pAAV-h*GCDH* transfer plasmid, a packaging plasmid (pRep2/Cap8 or pRep2/Cap9), and an adenoviral helper plasmid (pAd12). Viral particles were purified using CsCl gradient ultracentrifugation [35,36]. The titers of the purified vectors were determined via qPCR (CFX96 Real Time System, Bio-Rad Laboratories, Inc., Hercules, CA, USA). The final titers obtained were 2.35 × 10^13^ vector genomes (vg)/mL for rAAV2/8-h*GCDH* and 1.07 × 10^13^ vg/mL for rAAV2/9-h*GCDH*. Viral particle morphology was examined using transmission electron microscopy (EMUC7, Leica, Deer Park, IL, USA). In vitro expression was verified by infecting CHO-K1 and H4 cell lines at various multiplicities of infection (MOIs), followed by RT-PCR (T100™ Thermal Cycler, Bio-Rad Laboratories, Inc., Hercules, CA, USA) and qPCR analysis. The h*GCDH* primer (188 bp) forward, 5′-CCTCGTCATGCACCCTATCT-3′; reverse, 5′-TCCCATTGAGGGTGTAGCTC-3′ and the GAPDH primer as the internal control (100 bp): forward, 5′-CCCACTCCTCCACCTTTGAC-3′; reverse, 5′-CTGTTGCTGTAGCCAAATTCGT-3′ were used in both analyses.

#### 4.3.3. Transmission Electron Microscopy

Samples (rAAV-h*GCDH* vectors) were applied to a grid and embedded in a heavy metal salt solution. Semithin and ultrathin sections were prepared for ultrastructural observations. Sections were treated with 1% osmium tetroxide and embedded in epoxy resin. Semithin sections (0.5 μm) were stained with toluidine blue and observed using a microscope (BX51, Olympus, Tokyo, Japan). Ultrathin sections (70–80 nm) were counterstained with 0.25% lead citrate and 2% uranyl acetate and observed using a TEM (H-7650, Hitachi, Tokyo, Japan).

### 4.4. In Vivo rAAV-hGCDH Administration in GA1 Mice

All animal procedures were approved by the Institutional Animal Care and Use Committee (IACUC) of Ewha Woman’s University College of Medicine (EUM20-049) and conducted in accordance with the approved guidelines. The mice were housed in a temperature-controlled (21 ± 2 °C) and humidity-controlled (55 ± 5%) facility with a 12 h light/dark cycle with free access to a standard diet and water. For therapeutic evaluation, 5-week-old GA1 mice were randomly assigned to three experimental groups (*n* = 10/group), with WT mice serving as healthy controls. The sample size was determined based on previous GA1 mouse studies and pilot experiments demonstrating consistent biochemical differences. The rAAV vectors were suspended in 100 μL of phosphate-buffered saline (PBS), (WELGENE, Gyeongsan, Republic of Korea) and administered via tail vein injection at a dose of 5 × 10^11^ vg per mouse. The groups were designated as follows: (1) sham, (2) AAV8 (rAAV2/8-h*GCDH*), (3) AAV9 (rAAV2/9-h*GCDH*), and (4) WT. Therapeutic efficacy was assessed longitudinally by analyzing half of the animals in each group at 6 weeks post-injection and the remainder at 12 weeks.

#### 4.4.1. Western Blot Analysis

The brain and liver tissues were rapidly frozen in liquid nitrogen and then homogenized in PRO-PREP buffer on ice for 30 min. These samples were centrifuged at 13,000 rpm for 10 min at 4 °C, and equal amounts of protein from the supernatant were separated using the TGX Stain-Free™ FastCast™ Acrylamide Kit (BIO-RAD, Berkeley, CA, USA) in 1 × Tris/Glycine/SDS Buffer (BIO-RAD, Berkeley, CA, USA) at 80 V for 2 h. Protein transfer was performed with Trans-Blot Turbo (BIO-RAD, Berkeley, CA, USA) onto polyvinylidene fluoride membranes (BIO-RAD, Berkeley, CA, USA) using protocol 1.5 mm gel for 10 min. Membranes were incubated with primary antibodies, including anti-*GCDH* (1:1000; PA5-30040, Invitrogen, Carlsbad, CA, USA), a polyclonal antibody reactive with both human and mouse *GCDH*, according to the manufacturer’s specifications, and GAPDH (1:10,000) (AM4300, Invitrogen, Waltham, MA, USA), overnight at 4 °C. Then, membranes were incubated with secondary antibodies, Goat anti-rabbit IgG, HRP-linked Antibody (7074S, Cell Signaling, Danvers, MA, USA) and Goat Anti-Mouse IgG (H + L)-HRP Conjugate (1:10,000) (#1706516, Bio-Rad Laboratories, Inc., Hercules, CA, USA), and were incubated at a dilution of 1:10,000 for 1 h. The stained membranes were developed with Clarity Western ECL Substrate (BIO-RAD, Berkeley, CA, USA) and imaged with Amersham™ ImageQuant™ 800 (Cytiva Life Sciences, formerly GE Healthcare Life Sciences, Marlborough, MA, USA). The images were analyzed using ImageQuant TL (Version 10.0.261). Band intensities were quantified using Image J software (Version 1.49).

#### 4.4.2. Metabolic Profiling

The primary objective of metabolic profiling was to quantitatively assess GA across the various treatment groups to evaluate the impact of gene therapy on disease progression. This involved measuring GA concentrations in liver and brain tissue homogenates using highly sensitive and specific LC-MS/MS methods.

#### 4.4.3. Histological Analysis

Mice were transcardially perfused with PBS followed by 4% paraformaldehyde (Sigma-Aldrich, St. Louis, MO, USA). Brain sections were stained with hematoxylin and eosin to evaluate the general histopathology and detect subtle pathological changes.

### 4.5. Statistical Analysis

All quantitative data were analyzed using GraphPad Prism software (version 9.0) and expressed as the mean ± standard error of the mean. Statistical significance of the differences was determined using a Student’s *t*-test for GA concentration data. The results from the Western blot analysis and metabolic profiling were assessed for the significance of the differences by one-way analysis of variance (ANOVA) and Tukey’s post hoc multiple comparison test. *p* < 0.05 was considered significant.

## Data Availability

The original contributions presented in this study are included in this article/its Supplementary Materials. Further inquiries can be directed to the corresponding authors.

## Supplementary Materials

The following supporting information can be downloaded at: https://www.mdpi.com/article/10.3390/ijms27125569/s1.
