# Supplementary material for: Systemic AAV-hGCDH Gene Therapy Alleviates Glutaric Acid Accumulation and Attenuates Chronic Brain Vacuolation in a Novel Mouse Model of Glutaric Aciduria Type I
Source: Int J Mol Sci. 2026 Jun 20;27(12):5569. doi: 10.3390/ijms27125569 (PMC13299285; doi:10.3390/ijms27125569)
Supplement: Supplementary file 1 [file ijms-27-05569-s001.zip › ijms-4345004-supplementary.pdf]

**Supplementary Materials**

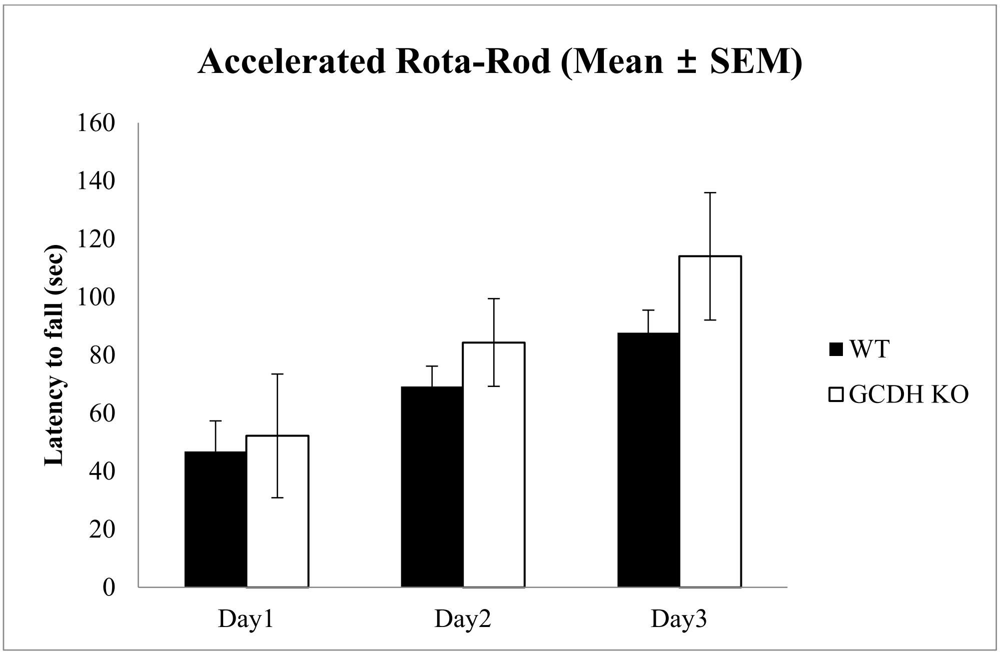


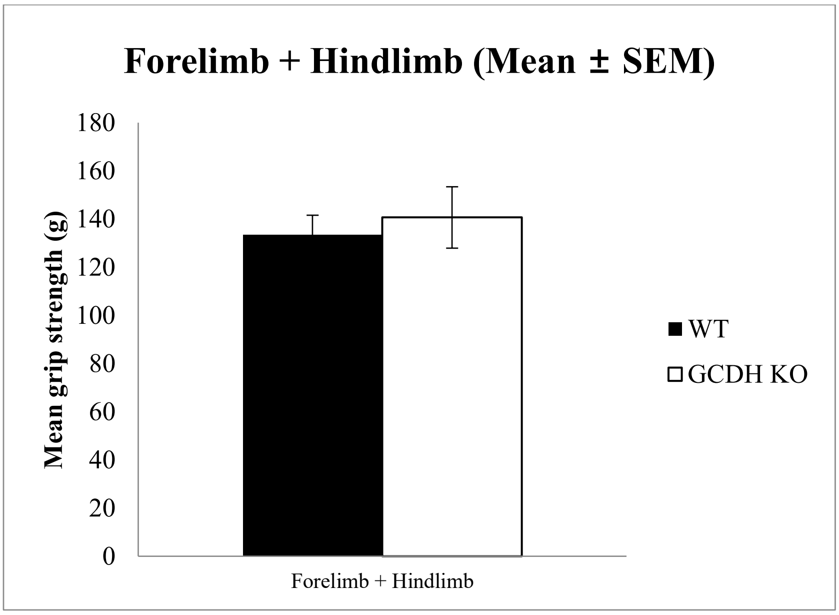


Figure S1. Behavioral phenotyping of GA1 mice under a standard diet.
To assess whether the chronic GA accumulation led to macroscopic functional deficits, we performed basic behavioral phenotyping. In the accelerated rotarod and grip strength tests, GA1 mice demonstrated motor coordination and muscle strength comparable to those of WT littermates. No statistically significant differences were observed between WT and GA1 mice in either test.

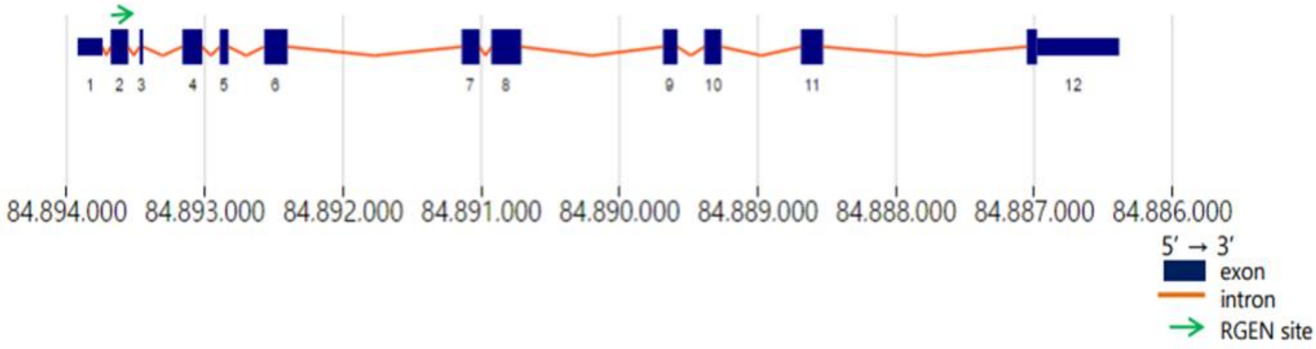


Figure S2. Deletion target sequence of mouse Gcdh gene

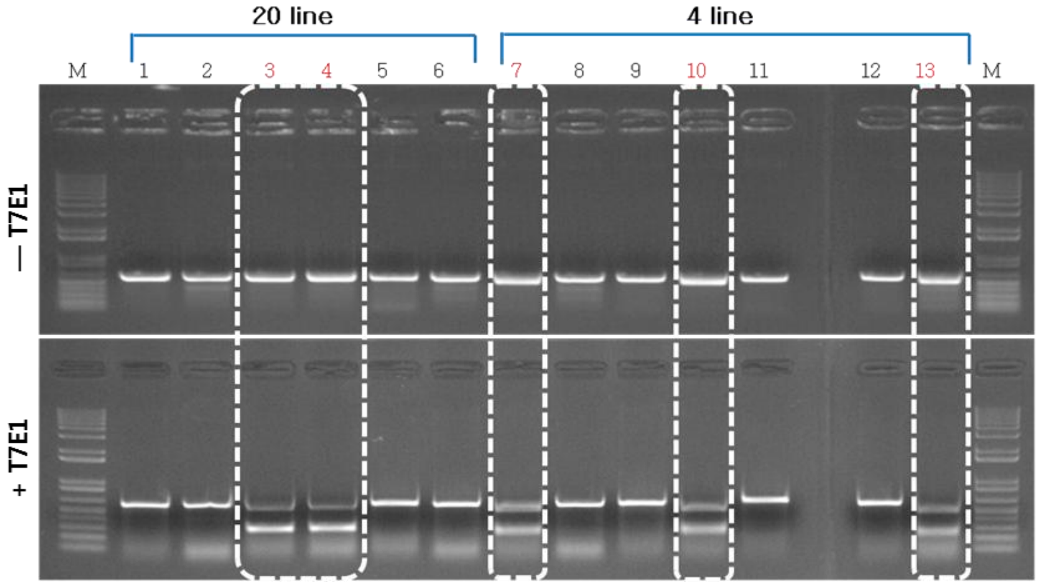


Figure S3. Genotyping of F0 generation mice

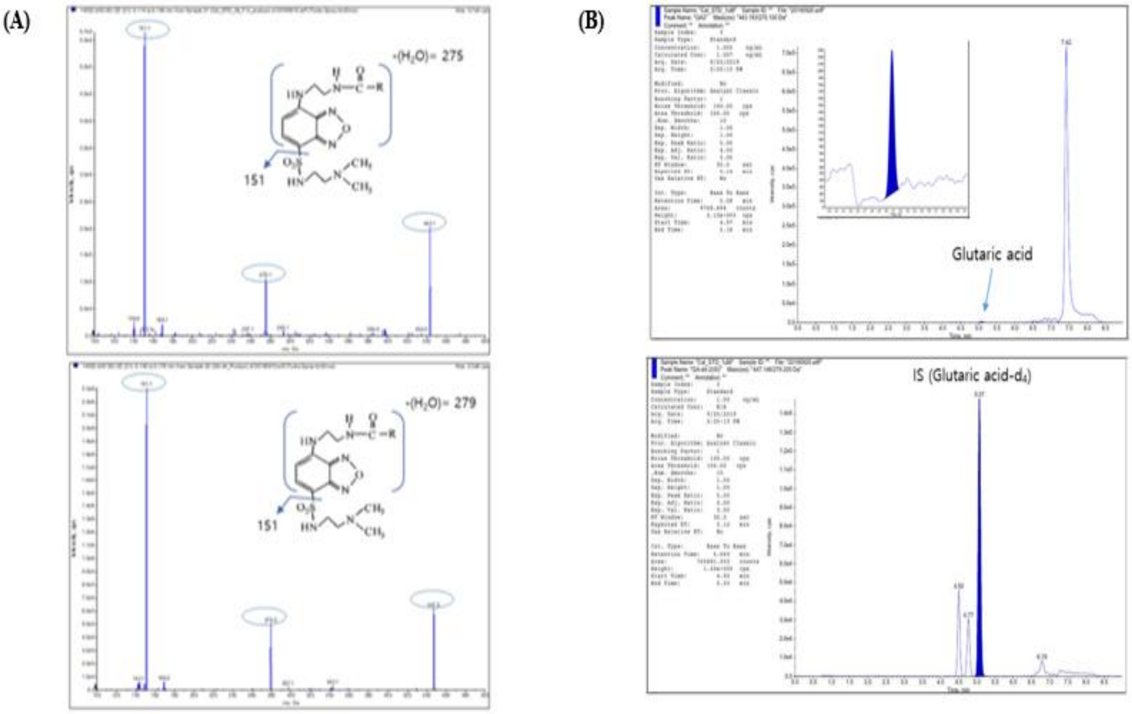


Figure S4. (A) MS/MS spectrum of glutaric acid derivative and internal standard (IS, glutaric acid-d4 derivative); glutaric acid derivative: m/z 443.183→275.100, IS (glutaric acid-d4 derivative): m/z 447.146→279.200 (B) Representative chromatogram of lowest limit of quantitation for glutaric acid (1 μM)

Sample Preparation and LC-MS/MS Conditions: GA was extracted from mouse urine, cerebrospinal fluid (CSF), brain, and liver tissues and derivatized prior to analysis. Ultra-performance liquid chromatography (UPLC) was employed to improve separation efficiency and sensitivity. Mass spectrometric detection was conducted in positive electrospray ionization (ESI+) mode using an Analyst® 1.7 software (AB Sciex Pte. Ltd., Singapore). Quantification was achieved using multiple reaction monitoring (MRM), monitoring precursor-to-product ion transitions of m/z 443.183 → 275.100 for the GA derivative and m/z 447.146 → 279.200 for the internal standard (GA-d4) (Figure S4).

Method Validation: The analytical method was validated according to standard guidelines. Key parameters assessed included selectivity, sensitivity, linearity, accuracy, precision, parallelism, and stability, all of which met acceptance criteria. Calibration curves demonstrated excellent linearity using a weighting factor of $1/x^2$ applied.

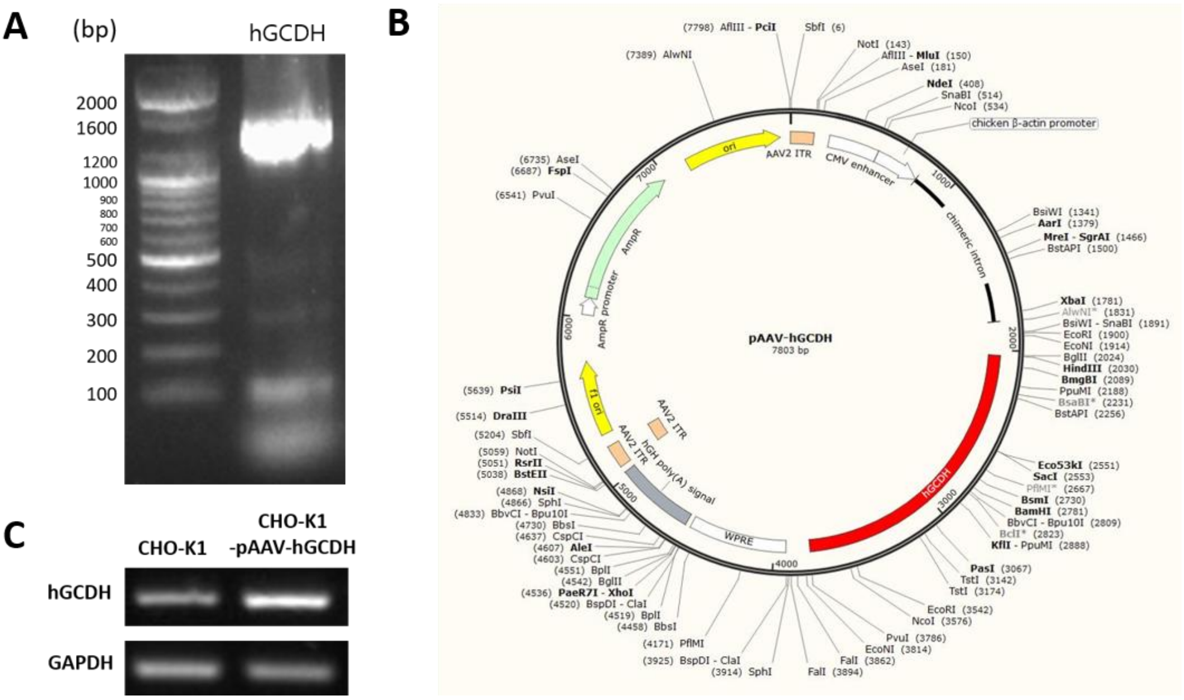


**Figure S5.** pAAV-hGCDH generation. (A) synthesis of hGCDH cDNA was confirmed. (B) pAAV-hGCDH plasmid map. (C) Transfection of pAAV-hGCDH into CHO-K1 cells and its expression was confirmed by RT-PCR.
